# Enhanced Spectral Tunability by Sub‐10 nm Nanogaps in Graphene‐Metal Hybrid Metasurfaces

**DOI:** 10.1002/advs.202506898

**Published:** 2025-07-30

**Authors:** Fei Han, Zaoyang Lin, Kacper Pilarczyk, Hongwei Tang, Guy A. E. Vandenbosch, Joris Van de Vondel, Xuezhi Zheng, Niels Verellen, Ewald Janssens

**Affiliations:** ^1^ IMEC Kapeldreef 75 Leuven 3001 Belgium; ^2^ Quantum Solid‐State Physics Department of Physics and Astronomy KU Leuven Celestijnenlaan 200D Leuven 3001 Belgium; ^3^ Department of Chemistry KU Leuven Celestijnenlaan 200F Leuven 3001 Belgium; ^4^ Faculty of Physics and Applied Computer Science AGH University of Science and Technology al. A. Mickiewicza 30 Kraków 30‐059 Poland; ^5^ WaveCoRE research group ESAT KU Leuven Kasteelpark Arenberg 10 Leuven 3001 Belgium; ^6^ Polariton‐driven Light‐Matter Interactions (POLIMA) University of Southern Denmark Campusvej 55 Odense 5230 Denmark

**Keywords:** electrically tunable metasurface, field enhancement, graphene transistor, metasurface, plasmonics, sub‐10 nm nanogaps

## Abstract

Electrically tunable graphene‐metal metasurfaces have emerged as a promising platform for precise control of free‐space light propagation. However, their resonance tuning range is limited by fabrication constraints, particularly by the achievable gap size between coupled antennas, which is the parameter that influences the device's performance most. In this work, this challenge is addressed by introducing a novel fabrication approach that combines traditional e‐beam lithography with physical vapor deposition of an additional thin metal layer and subsequent ion milling. Incorporating an Al_2_O_3_ etch‐stop layer allows to overcome the ≈20 nm gap size limitation of conventional methods. Using this approach sub‐10 nm gaps can be fabricated reliably and the tuning range of metasurfaces operating in the mid‐infrared is increased from 0.50 to 0.77 µm, together with an enhancement in the maximum modulation depth from 45% to 59%. The better performance is attributed to stronger field enhancement in the reduced nanogap. This work is a critical step toward widely tunable mid‐infrared metasurfaces, with potential applications in spatial light modulators, surface‐enhanced Raman spectroscopy, and quantum photonics.

## Graphical abstract

A tunable mid‐infrared metasurface integrating graphene and metal antenna arrays with sub‐10 nm nanogaps is presented, overcoming the ≈20 nm limit of conventional fabrication. The enhanced spectral tuning range and modulation depth are enabled by combining electron‐beam lithography, thin metal deposition, and directional ion milling to precisely define ultra‐narrow gaps for efficient light–matter interaction.

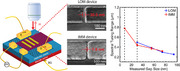

## 1 Introduction

Metasurfaces are engineered 2D structures composed of arrays of subwavelength‐scaled elements, known as meta‐atoms. These structures enable extraordinary control over the phase, amplitude, and polarization of light.^[^ ^1^ ^]^ While the optical properties of traditional passive metasurfaces are fixed during fabrication, there is a growing demand for more functionalities through dynamic control. As a result, active metasurfaces are gaining interest in the field of nanophotonics, with applications such as optical modulators,^[^ ^2^, ^3^ ^]^ dynamic wavefront manipulators,^[^ ^4^, ^5^ ^]^ and biosensors.^[^ ^6^, ^7^, ^8^ ^]^

Among the various designs considered for tunable metasurfaces, graphene‐metal hybrid metasurfaces have attracted considerable attention due to the exceptional tunability of graphene's electronic conductivity. A widely used platform for dynamically controlling graphene's optical and electrical properties is the graphene field‐effect transistor (GFET), where graphene serves as the conductive channel between source and drain electrodes, and its carrier density is modulated by an applied gate voltage.^[^ ^9^, ^10^, ^11^ ^]^ However, one of the primary challenges in integrating graphene into tunable metasurfaces is its low optical absorption (≈2.3%) in the visible to infrared spectral range, which limits the optical modulation capabilities.^[^ ^12^, ^13^, ^14^, ^15^, ^16^ ^]^ A promising approach to overcome this limitation involves paired antenna arrays, which confine light in the plane of graphene, thereby enhancing light‐matter interactions and improving spectral tunability.^[^ ^14^, ^17^, ^18^, ^19^, ^20^ ^]^ Such enhancement strategies can also be extended to different spectral ranges^[^ ^21^ ^]^ or combined with alternative tuning mechanisms.^[^ ^22^, ^23^ ^]^ Nonetheless the tuning range remains limited, unless chemical doping is used^[^ ^17^ ^]^ or large voltages are applied,^[^ ^24^, ^25^ ^]^ which hinders practical use. An alternative and more practical solution to further increase optical tunability is the creation of nanogaps between the paired metal antennas with strong local field enhancement.^[^ ^26^, ^27^, ^28^ ^]^

Bottom‐up methods can be used to realize a nanometer‐scale cavity, as was shown by spacers consisting of single molecules of well‐defined orientation,^[^ ^29^, ^30^, ^31^ ^]^ such as methylene blue in cucurbit[*n*]uril.^[^ ^30^ ^]^ A chemical fabrication technique was used to create a 0.9 nm thick molecular spacer between gold nanoparticles and a gold mirror, obtaining a maximal electric field enhancement by a factor 400, enabling strong coupling between visible light and individual quantum emitters at room temperature.^[^ ^30^ ^]^ However, integrating bottom‐up fabricated nanostructures with existing optoelectronic devices or electrical circuits presents significant challenges.^[^ ^32^, ^33^ ^]^ Additionally, the reproducibility and scalability of these methods are limited. Top‐down methods, such as extreme ultraviolet lithography,^[^ ^34^, ^35^, ^36^ ^]^ atomic layer lithography,^[^ ^37^, ^38^, ^39^, ^40^ ^]^ nanoimprint lithography,^[^ ^41^, ^42^, ^43^ ^]^ and the ‘sketch and peel’ strategy,^[^ ^44^, ^45^ ^]^ show promise to overcome these limitations, but they are relatively expensive or guarantee only limited throughput.^[^ ^46^, ^47^, ^48^, ^49^ ^]^ Efforts have also been made to leverage a multiple patterning technique to reduce pitch line width by creating sidewall spacers on a core, as in the self‐aligned double patterning technique.^[^ ^50^ ^]^ Nevertheless, high costs remain a challenge due to the required additional lithography steps.^[^ ^51^ ^]^ Hence, the highly reproducible, accurate, and cost‐effective fabrication of paired metallic antennas with sub‐10 nm nanogaps remains a critical technical challenge.

In this work, we present a GFET integrated with plasmonic antennas featuring sub‐10 nm nanogaps, whose resonance frequency can be actively modulated in the mid‐infrared range by an applied gate voltage. Uniform sub‐10 nm nanogaps are fabricated by reducing the conventional gap size through Physical Vapour Deposition (PVD) of a thin metal layer and subsequent ion milling. The relationship between optical tunability and gap size is experimentally investigated. By incorporating a paired antenna array with ≈10 nm nanogaps into the GFET, the resonance wavelength can be actively modulated in a broader range, and a higher modulation depth can be reached. The approach provides a robust fabrication method for optical devices requiring precise nanogap control, which is essential for increasing the applicability of metasurfaces containing 2D materials.

## 2 Results and Discussion

### 2.1 Reliable Fabrication of the Metasurface with Sub‐10 nm Nanogaps

GFETs integrated with paired antenna arrays fabricated via a lift‐off process have been studied earlier.^[^ ^14^ ^]^ Their mid‐infrared resonance was shown to be tunable through an applied bias voltage because of the enhanced light confinement within the gaps between the paired antennas. The gap size that could be obtained through conventional resist‐based patterning was limited to 20 nm.

**Figure 1:**
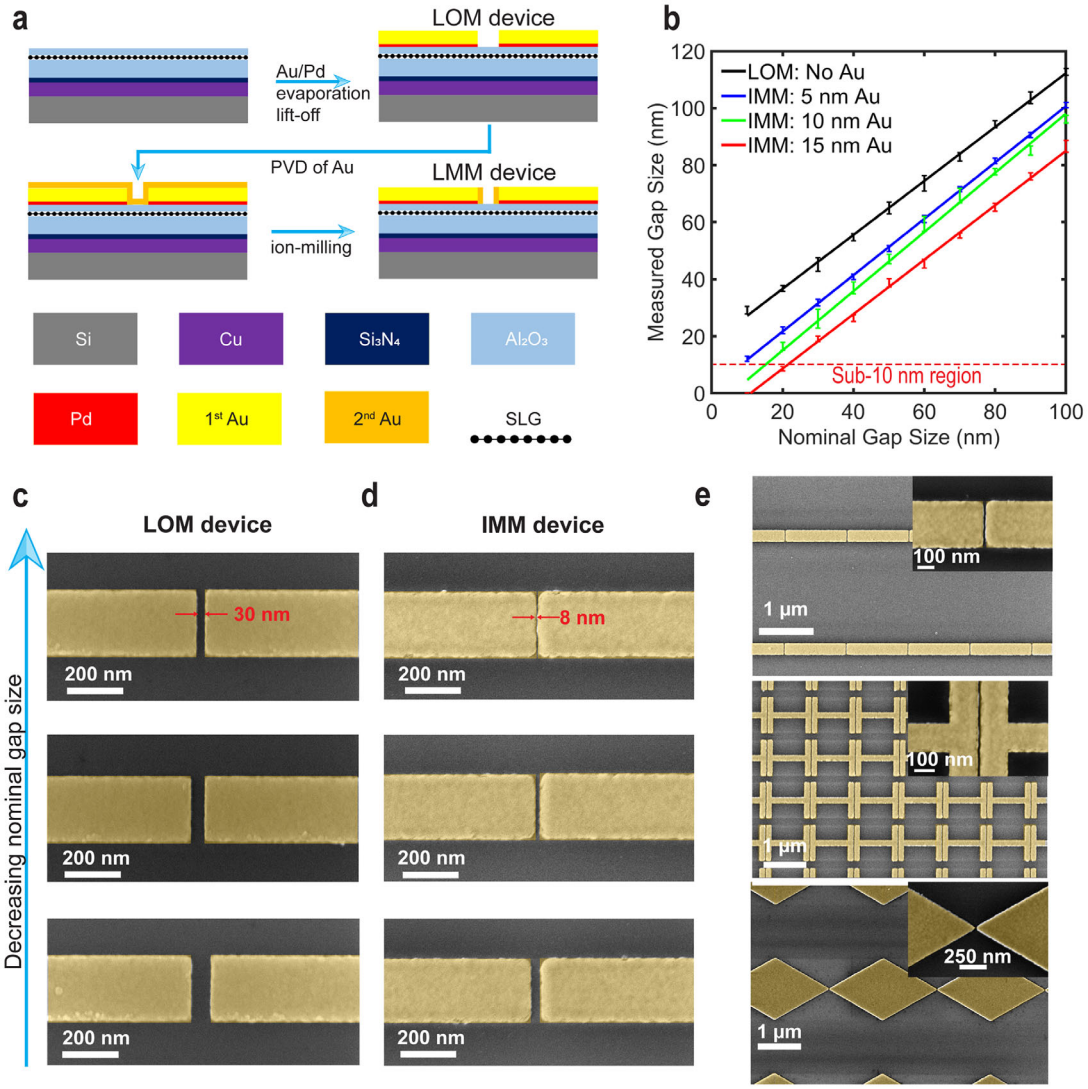
a) Schematic of the fabrication process illustrating the preparation of arrays of antennas that are separated by nanogaps. b) Measured nanogap size as a function of the nominal gap size for different thicknesses of the PVD Au layer. SEM images of c) LOM and d) IMM metasurfaces for different nominal gap sizes (scale bar, 200 nm). For the IMM metasurfaces a PVD Au thickness of 15 nm was used. e) Demonstration of the realisation of sub‐10 nm gaps in different morphologies: rectangular‐shaped (top), ‘I’‐shaped (middle), and triangular‐shaped (bottom) gold antenna arrays. The insets contain zoomed‐in images around a single nanogap.

A new process flow that allows to make smaller nanogaps between the coupled antennas, is shown in **Figure** **1**a. First, 4 nm Al_2_O_3_ is deposited via Atomic Layer Deposition (ALD) on the substrate, serving as an etch stop layer during ion milling. The substrate incorporates a stack of Al_2_O_3_ (300 nm) / Si_3_N_4_ (15 nm) / Cu (250 nm), which forms the bottom part of a Fabry–Pérot (FP) cavity (see Methods for details). The FP cavity is introduced to enhance light confinement within the device. Next, e‐beam lithography (EBL) is employed to define the paired antenna arrays with nanogaps, followed by the deposition of a 30 nm thick Au layer with a 5 nm Pd adhesion layer and subsequent lift‐off. A metasurface that is formed this way is called a Lift‐off Metasurface (LOM). To obtain smaller nanogaps, the fabrication continues with a new step. An additional gold layer is added on top of the LOM using PVD. Due to the anisotropic nature of the PVD, this adlayer conformally covers also the vertical sidewalls of the antennas. Finally, the Au layer at the bottom of the nanogaps is selectively removed using ion milling. This yields the metasurfaces that will be further referred to as ion‐milled metasurface, IMM.

**Figure 2:**
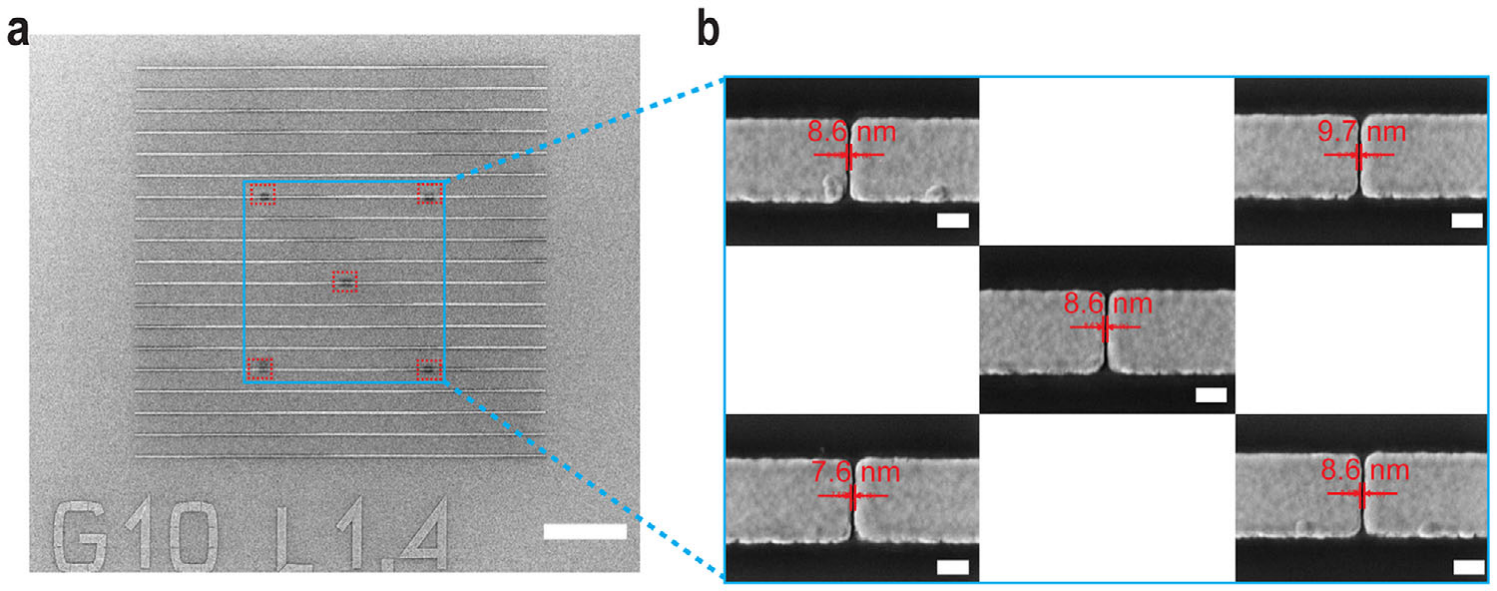
a) SEM image at 2000× magnification of the fabricated sub‐10 nm gap metasurface (scale bar 10 µm). b) Zoomed‐in SEM images at 250,000× magnification showing sampling locations for the gap size measurements (scale bar 100 nm).

To evaluate the quality of the fabrication process, we employ a point‐by‐point sampling method for both LOM and IMM devices, measuring gap widths using scanning electron microscopy (SEM). **Figure** **2**a shows a typical fabricated metasurface with an area of 60 × 60 um^2^. To assess the uniformity of the fabrication, SEM imaging was conducted at multiple locations, as indicated by the red dashed squares that are separated more than 10 µm from each other to demonstrate uniformity (Figure 2b).

The measured average gap sizes together with the standard deviation are plotted against the nominal gap sizes (i.e. the design dimension in the lift‐off procedure) for different thicknesses of the PVD Au layers in Figure 1b. The data indicates successful fabrication of sub‐10 nm nanogap when the nominal gap size is 20 nm and the PVD Au film is 15 nm thick. The measurements also show that the gap size of the LOM device is ≈15 nm wider than the nominal value. This is due to the proximity effect, which occurs when adjacent features are so close that scattered electrons from the primary beam unintentionally expose surrounding resist areas, resulting in larger gap sizes than intended.^[^ ^52^, ^53^ ^]^ For the same nominal gap size, a thicker PVD Au layer results in a smaller fabricated gap size, with an optimal thickness of 15 nm to achieve the sub‐10 nm nanogaps. Comparison of the SEM images reveals an increased edge roughness of the IMM metasurfaces (Figure 1d) compared to the LOM devices (Figure 1c). This is attributed to the combined effects of gold deposition and ion milling.

The new fabrication process can be extended to other nanoscopic morphologies that require sub‐10 nm nanogaps. To demonstrate the versatility, triangular, rectangular, and I‐shaped nanostructures were fabricated using the same method (Figure 1e). All these structures exhibit uniformly≈10 nm nanogaps over a 60 × 60 µm^2^ area, confirming the reliability and scalability of the approach.

The reflectance spectra of the fabricated metamaterials with different gap size were investigated by FTIR spectroscopy. It was found that all metasurfaces show a clear dipolar resonance, with the resonance wavelength shifting to the red for narrower gaps, which can be attributed to the enhanced coupling (see Section SI, Supporting Information for details).

### 2.2 Electrical Characterization of the GFET Devices

**Figure 3:**
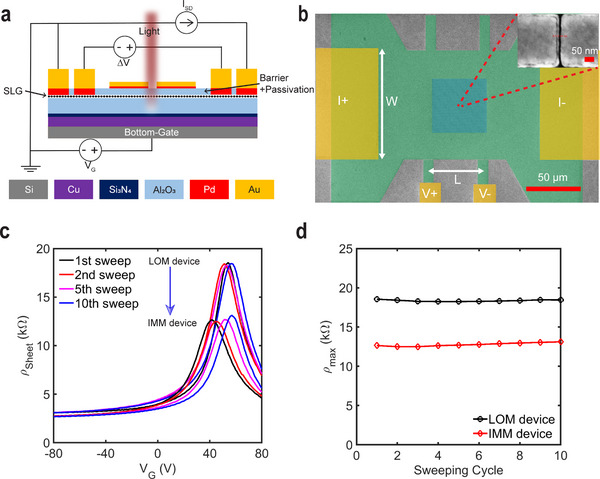
Configuration and electric transport characteristics of the GFET devices. a) Schematic representation of the electrical setup for the GFET device based on a five‐terminal architecture. b) False colored SEM image of the fabricated device (green: graphene, yellow: Au contacts, blue: area with the metal antenna arrays). Scale bar, 50 µm. Inset: A zoomed‐in SEM image showing a sub‐10 nm nanogap between two rectangular antennas (scale bar, 50 nm). c) Measured graphene sheet resistivity of the LOM and IMM devices as a function of gate voltage for successive sweeping cycles. d) Evolution of *ρ*_*max*_ for the LOM and IMM devices with increasing sweeping cycle. The LOM device has a measured gap size of ≈30 nm, and the IMM device has a measured gap size of ≈8 nm after processing with the additional 15 nm PVD Au layer deposition and following ion‐milling step.

Since the rectangular IMM devices exhibit a good uniformity and have been demonstrated to provide a large field enhancement,^[^ ^54^ ^]^ they were selected to investigate the spectral tunability across different gap sizes. To characterize the electrical transport properties of these devices, we integrated the proposed multilayer structure with a GFET architecture. Four‐point probe measurements were employed, as illustrated schematically in **Figure** **3**a. The GFETs were fabricated in a back‐gate configuration, and the resistivity is measured via the current and voltage contacts shown in Figure 3b. The graphene sheet resistivity is calculated using the following relation:

(1) $$\rho_{Sheet}=W\;\Delta V/L\;I_{SD},$$

where Δ*V* is the measured voltage between the middle contacts, *I_SD_* is the applied source‐drain current (set to 1 µA), and *W* (100 µm) and *L* (50 µm) are the width and length of the probed rectangular graphene strip, respectively.

According to electromagnetic perturbation theory, reducing the physical distance between the nanoantenna and graphene enhances the influence of variations in graphene's dielectric function on the optical response of the system, thereby improving the tunability of the reflection spectrum.^[^ ^55^ ^]^ Therefore, the Al_2_O_3_ layer between the nanoantenna and graphene should be as thin as possible, while still ensuring effective insulation. If the latter condition is not met, i.e., if the Al_2_O_3_ layer becomes too thin, a deterioration of the device's performance can be observed. An Al_2_O_3_ layer thinner than 2 nm can be penetrated easily during the ion milling, even at a relatively low etching rate, directly damaging the graphene channel.^[^ ^56^ ^]^ In addition, a thin Al_2_O_3_ layer may suffer from incomplete coverage, leaving pinholes and defects that can yield unwanted and uncontrolled doping caused by the ambient gas adsorption, the presence of dangling bonds or Xe ion‐induced changes from the milling process.^[^ ^57^ ^]^ To balance these effects, it was opted to deposit a 4 nm Al_2_O_3_ edge stop layer onto the graphene.

We characterized and compared the transport properties of the GFETs with the Al_2_O_3_ adlayer before and after milling to assess its impact. Figure 3c presents the measured sheet resistivity of the LOM and IMM devices as a function of gate voltage (*V_G_*) over multiple sweeping cycles. Both the LOM and IMM devices exhibit a charge neutrality point (CNP) ≈60 V, indicating that holes dominate the charge transport. Even in the presence of the 4 nm Al_2_O_3_ etch stop layer, the maximum resistivity decreases ≈30% after milling. This decrease is most likely caused by the formation of defects or pinholes in the thin Al_2_O_3_ layer (see Section SIII, Supporting Information for details). Their presence increases the influence of ambient gas adsorption and the likelihood of Xe ion‐induced changes, resulting in uncontrolled doping.^[^ ^58^, ^59^ ^]^ The former contribution can also be identified through the shift in the charge neutrality point (CNP) over subsequent sweeps – a behavior previously observed for uncapped GFETs. The extracted maximum resistivity (*ρ_max_*) as a function of the cycle number is plotted in Figure 3d for the LOM and IMM devices. Although *ρ_max_* is lower for the IMM devices, both devices demonstrate a stable minimal number of charge carriers in graphene over consecutive sweeps.

It is worth noting that the maximal applied bias voltage (≈80 V) is relatively high compared to modern CMOS standards, primarily due to the increased Al_2_O_3_ required to form the FP cavity. This voltage requirement could be reduced by two main approaches: i) decreasing the equivalent oxide thickness (EOT) using high‐k dielectrics such as HfO_2_ or Ta_2_O_5_,^[^ ^60^ ^]^ and ii) employing a thin high‐k top‐gate dielectric to bring the operating voltage down to a few volts. However, since conventional metal gates are not transparent in the infrared region, implementing low‐voltage gating often requires alternative designs to maintain optical transparency. This includes the use of transparent top gates—such as a second graphene layer—or other infrared‐transparent conductors, enabling electrostatic modulation without compromising optical transmission.^[^ ^61^, ^62^ ^]^

### 2.3 Active Reflectance Tuning of the Mid‐Infrared Metasurfaces

To quantify the dependence of spectral tunability on the gap size, the reflectance spectra of LOM and IMM devices were measured for different values of the applied gate voltage. The spectra were normalized by the reflectance spectrum of a sample with an identical Al_2_O_3_/Si_3_N_4_/Cu substrate that was coated with a 200 nm Au layer (see details in the Experimental Section).

The spectral tuning of the GFETs can be quantified by:

(2) $$\Delta \lambda_{res}=\lambda_{res,1}-\lambda_{res,0},$$

where *λ*_*res*,*0*_ is the resonance wavelength at the CNP of the GFET, and *λ*_*res*,*1*_ is the resonance wavelength when the device is gate‐biased at the maximum applied voltage of −80 V. The resonance tuning follows the expected dependence on graphene's charge carrier concentration. As the applied gate voltage increases, the charge carrier concentration rises, leading to an increase in graphene's permittivity and a corresponding blue shift in the spectra.^[^ ^17^ ^]^

**Figure 4:**
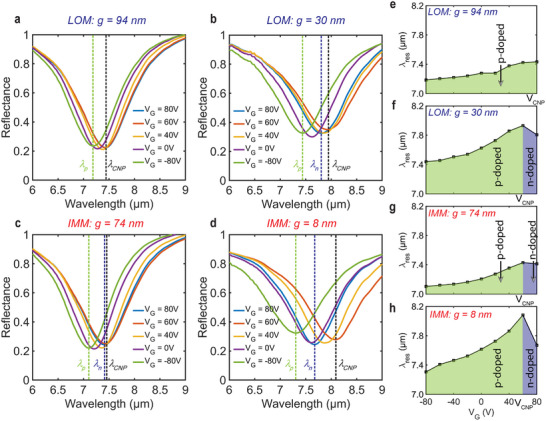
FTIR reflectance spectra for different values of the back‐gate voltage *V*_*G*_ of a) LOM with gap size *g* = 94 nm, b) LOM with *g* = 30 nm, c) IMM with *g* = 74 nm, and d) IMM with *g* = 8 nm. The vertical dashed lines mark *λ*_*CNP*_, *λ*_*p*_, and *λ*_*n*_, which are the resonance wavelengths at the charge neutrality point, at p‐doped and n‐doped graphene, respectively. Dependence of the resonance wavelength on the back‐gate voltage of e) LOM with *g* = 94 nm, f) LOM with *g* = 30 nm, g) IMM with *g* = 74 nm, and h) IMM with *g* = 8 nm. Regions shaded green (blue) correspond to the GFETs operating in the p‐doped (n‐doped) regime.

**Figure** **4**a–d shows the reflectance spectra of the LOM and IMM devices under different back‐gate bias voltages. To visualize the spectral tunability, the extracted resonance wavelengths at various *V_G_* values are plotted in Figure 4e–h. For the 94 nm gap LOM device, the reflectance spectra exhibit a blue shift of 0.25 µm as the back‐gate voltage is decreased from 80 to – 80 V (Figure 4a,e), while the 30 nm gap LOM device exhibits a significantly larger blue shift of 0.50 µm for a voltage change from the *V*_*CNP*_ at 60 V to the most p‐doped situation at *V*_G_ = – 80 V (Figure 4b,f).

The tuning range is found to be larger for graphene‐metal hybrid metasurfaces with smaller gaps. The *g* = 74 nm IMM device exhibits a blue shift of 0.33 µm as the back‐gate voltage is decreased from *V*_*CNP*_ of 60 to – 80 V, while the 8 nm IMM device shows the largest blue shift of 0.77 µm for the same back‐gate voltages. Compared to the LOM devices, the spectral tunability of the IMM devices increases by 32% and 54% for nominal gap sizes of 80 and 30 nm, respectively.

To support the experimental results, we performed finite‐difference time‐domain (FDTD) simulations of the reflectance spectra for metasurfaces with gap sizes of 100, 80, 30, and 10 nm for different Fermi level energies. Those spectra are shown in Figure S5a–d (Supporting Information). The simulated trends are in good agreement with the experimental observations. In particular, they confirm that metasurfaces with smaller gap sizes exhibit a larger spectral tuning range.

The improved spectral tunability primarily results from the increased field enhancement associated with the reduced gap size. For the same nominal gap size, the actual gap sizes of the IMM devices are smaller than those of the LOM devices. Notably, the metasurfaces with sub‐10 nm nanogaps, showing the larger tunability, have the strongest field enhancement. This confirms the initial assumption that achieving sub‐10 nm gaps is crucial for improving the performance of GFET‐based modulators.

The spectral tunability can be understood using the equivalent circuit model (ECM) as described in previous work.^[^ ^14^ ^]^ In this model, the antenna arrays are part of the circuit, including an inductor *L_s_*, a capacitor *C_s_*, and a resistor *R_s_*. The spectral frequency shift is given by:^[^ ^14^ ^]^

(3) $$\Delta \omega \approx \frac{1}{2\omega_{0}L_{s}C_{mx}\left(\omega_{0}^{2}L_{G}C_{mx}-\frac{C_{mx}}{C_{mz}}-1\right)},$$

where the original resonant frequency is *ω_0_*, the frequency shift is given by Δ*ω*, the capacitance of the mutual coupling between the antennas is *C_mx_*, the capacitance of the additional Al_2_O_3_ layer is given by an in‐series capacitance *C_mz_*, and *L*_*G*_ is the inductance of the graphene. *L*_*G*_ can be approximated as:^[^ ^14^ ^]^

(4) $$L_{G}=\frac{g}{{\omega_{0}}^{2}\varepsilon_{0}\left(-\varepsilon_{G}^{\prime }\right)wt_{G}},$$

with *g* the gap size between the antennas, *w* is the width of the graphene, $\varepsilon_{G}^{\prime }$ is the real part of the graphene permittivity, and *t_G_* is the thickness of the graphene. For a given charge carrier concentration modulation capability in the graphene, *L_G_* decreases linearly with the reduction of the gap size between the antennas, which increases the spectral tunability.

**Figure 5:**
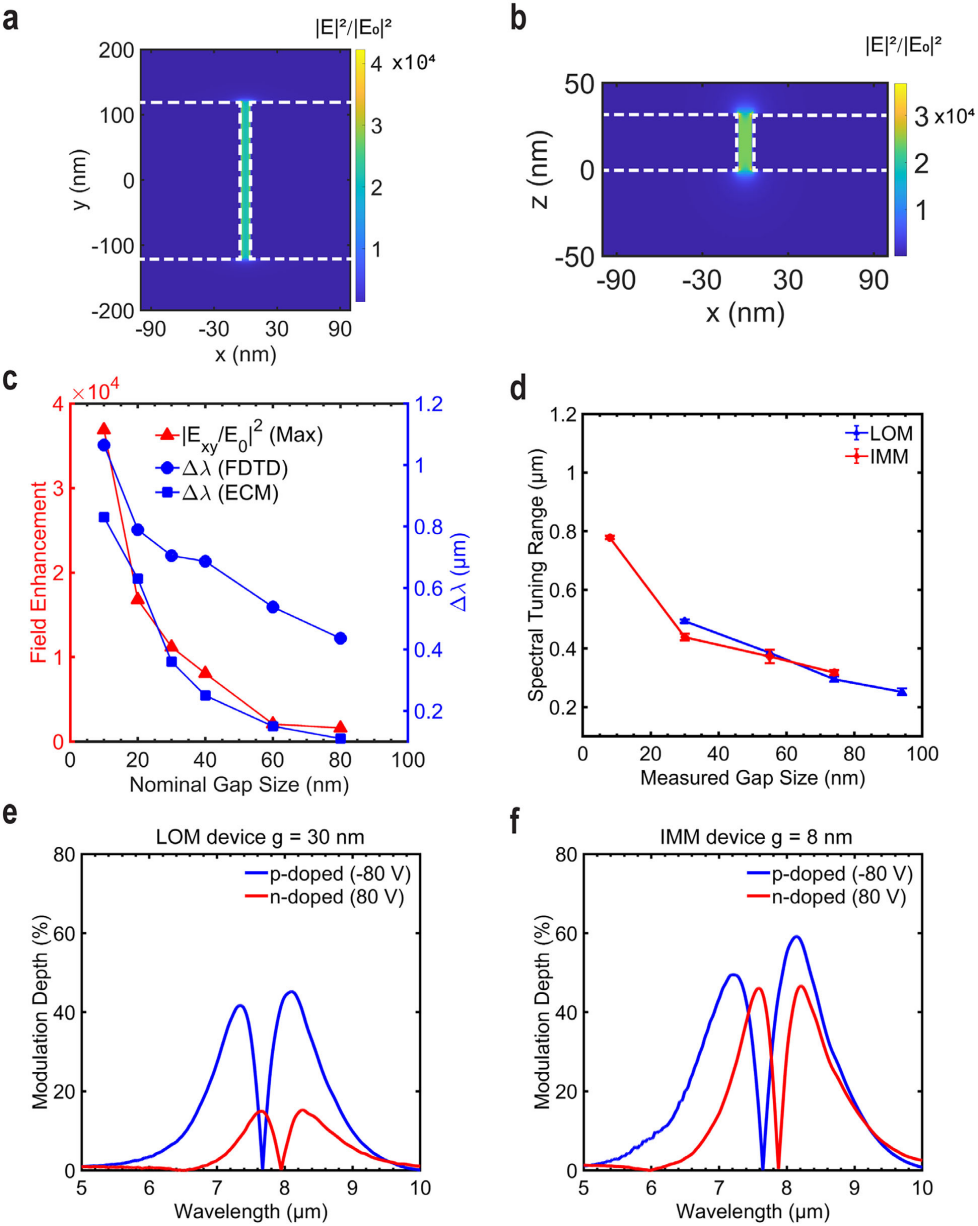
The simulated field enhancement distribution in the a) x‐y plane and b) x‐z plane around the nanogap of the rectangular metasurface with a 10 nm gap size. c) Simulated field enhancement and spectral tuning range according to the FDTD simulation and tuning range according to the ECM as a function of the gap size, with graphene placed on a 4 nm Al_2_O_3_ layer. d) Measured spectral tuning range of the fabricated LOM and IMM devices as a function of their actual gap size. Measured reflectance modulation depth for the OFF‐ON transition of e) the LOM device with *g* = 30 nm and f) the IMM device with *g* = 8 nm as a function of the wavelength. Blue solid line: the device working in the p‐doped graphene regime; red solid line: the device working in the n‐doped graphene regime.

To further explore the physical origin of the increased tunability, FDTD simulations were used to investigate the field enhancement in the designed metasurfaces for different *g* values. For a proper comparison of the tunability across different resonances, the antenna length is fixed at 2.0 µm. The simulated electric field distribution within the antenna arrays for 10 nm nanogaps is presented in **Figure** **5**a,b, showing a maximum field enhancement in the x‐y plane of ≈4 × 10^4^. As shown in Figure 5c (red circles) the field enhancement increases strongly as the antenna gap size decreases. It shows that despite the presence of the 4 nm Al_2_O_3_ barrier layer on top of the graphene, there is a significant field enhancement within the nanogap, facilitating strong light‐graphene interactions.

The spectral tunability *∆λ* was simulated by calculating the Fermi level range (from 0 to 0.25 eV) based on the charge carrier concentrations, which are approximated from the back‐gate voltages used in the experiments. The detailed calculation was described in the previous study.^[^ ^14^ ^]^ The simulated reflectance spectra are shown in Figure S5 (Supporting Information) and discussed in Section SIV (Supporting Information). The FDTD simulations predict a spectral tuning range of ≈1.1 µm for a metasurface with 10 nm nanogaps (Figure 5c, blue circles), which closely matches the experimental result for the IMM device with a measured gap size of 8 nm. Additionally, the more conceptual ECM (Figure 5c, blue squares) qualitatively reproduces the gate‐controlled resonance shift and its dependence on the size of the nanogaps. The values are of the same order of magnitude but there is no quantitative agreement, which is according to expectations in view of the simplifying assumptions inherent to the ECM. The ECM does not fully capture the complex field distributions and the plasmonic interactions present in nanogap regions.^[^ ^63^ ^]^ The experimental results are summarized in Figure 5d. Four measurements were conducted for each device, with the average values and the standard deviation depicted to demonstrate the reproducibility. The difference between the experimental (0.8 µm) and simulated (1.1 µm) tunability narrow‐gap IMM device can be attributed to two factors. First, additional doping and the introduction of residual charge traps from ion milling reduce the maximum resistivity, indicating slight graphene degradation. This issue can, in future work, be mitigated by improving the seeding technique and using a more conformal ALD deposition process. Second, in the simulation, we assumed the applied gate voltage of −80 V corresponds to the Fermi level shift of 0.25 eV (see Section SIV, Supporting Information), which introduces some uncertainty on the permittivity of the graphene under the applied bias. This uncertainty could be reduced by direct measurements of the bias dependence of the graphene conductivity.^[^ ^64^ ^]^

To evaluate the device's performance as a mid‐infrared modulator, we use modulation depth (MD) as a figure of merit. It is defined as:

(5) $$\mathrm{MD}\left(\lambda \right)=\left|\Delta R\left(\lambda \right)\right|/\max\left[\left(R_{\mathrm{on}}\left(\lambda \right);R_{\mathrm{off}}R_{\mathrm{on}}\left(\lambda \right)\right)\right]\times 100,$$

where Δ*R*(*λ*) is the change in reflectance, with *R_ON_* (*λ*) and *R_OFF_* (*λ*) representing the reflectance values at the chosen wavelengths in the ON and OFF states, respectively.^[^ ^14^, ^24^, ^65^ ^]^ As seen in Figure 5e,f, both the LOM and IMM devices exhibit p‐doped and n‐doped modulation, with the maximum MD increasing from 45% to 59% if the gas size in the devices decreases from 30 nm to 8 nm. This result demonstrates the effectiveness of the proposed technique in enhancing modulation performance. These trends are further supported by FDTD simulations (Figure S6, Supporting Information), which predict the reflectance changes for metasurfaces with gap sizes of 30 and 10 nm. The simulated maximum modulation depth reaches unity due to the idealized perfect absorption design, whereas the experimental maximum MD is lower due to fabrication limitations and variations in material properties.

To benchmark the fabricated hybrid metasurface against recent advances in mid‐infrared modulators, a comparative summary is provided in **Table** **1**. This table outlines essential performance metrics, including device design, order of the layers, the feature critical dimension (CD) of the design, the maximal modulation depth (MD), and modulation efficiency, which is defined as the ratio of maximum MD to the corresponding driving voltage. The modulation efficiencies achieved with our devices—0.56%/V for the 30 nm nanogap and 0.74%/V for the 8 nm nanogap—exceed those of most previously reported mid‐infrared modulators, except for the waveguide‐integrated antenna design in Ref. [66] which reaches 1.19%/V. This higher efficiency results from enhanced light–graphene coupling enabled by the deep‐subwavelength nanogaps of the V‐shaped antennas, which also increased fabrication complexity.^[^ ^66^ ^]^

**Table 1:** Comparison of the performance of graphene‐based devices for tunable metasurfaces.

Device design	Order of layers	Feature CD	Max. MD	Efficiency [%/V]	Refs.
Au rods + graphene	Au/Cr/graphene/ 90 nm SiO_2_/p^++^ Si	70 nm	17%	0.43	[67]
Waveguide antenna	Au/Pd/graphene 300 nm AlO_x_/Al	30 nm	95%	1.19	[66]
Au grating + graphene nanoribbon	Au/Ti/graphene/SiO_2_/ SiN_x_/Au	50 nm	44.6%–95.9%	0.17–0.37	[68]
Au ribbon/disk + graphene ribbon/disk	Au/Cr/graphene/BaF_2_	90 nm	24%	0.12	[69]
Au paired antennas + graphene	Au/Pd/graphene/ 300 nm SiO_2_/p^++^ Si	> 20 nm	36%	0.18	[14]
Graphene + Si rods	Ion gel/graphene/Si/SiO_2_	400 nm	28.9%	0.15	[70]
Graphene resonator	Graphene resonator/285 nm SiO_2_/Si	50 nm	11%	0.18	[71]
Au paired antennas + graphene	Au/Pd/graphene/Al_2_O_3_/ Si_3_N_4_/Cu	30 nm	45%	0.56	this work
Au paired antennas + graphene	Au/Pd/graphene/Al_2_O_3_/ Si_3_N_4_/Cu	8 nm	59%	0.74	this work

## 3 Conclusion

In summary, we demonstrated that the combination of the lift‐off, the addition of the thin metallic layer by physical vapor deposition and subsequent ion‐milling processes provides a reliable fabrication procedure for uniform sub‐10 nm nanogaps between paired antennas. In contrast, the commonly employed methods are limited to gap sizes above 20 nm, which restricts the field enhancement magnitude and, consequently, limits the tuning range of optical systems. With this approach we could increase the tuning range from 0.50 µm to 0.77 µm for a 8.0 µm resonance. Additionally, the maximum modulation depth increases from 45% to 59%.

Further improvements in the MD and the tuning efficiency can be pursued by either enhancing electrostatic tunability or optimizing optical absorption at the resonance. Larger tunability may be achieved using a dual‐layer graphene structure combined with a thin, high‐κ dielectric top gate, which significantly increases charge carrier density under bias and thereby boosts graphene's optical absorption, leading to a higher MD.^[^ ^61^, ^62^, ^72^ ^]^ Alternatively, maximizing absorption near the resonance wavelength can be realized by approaching the critical coupling regime through precise tuning of the dielectric layer thicknesses and other structural parameters.

The presented method can be extended to other systems involving metallic metasurfaces. The study not only offers a viable solution for tunable mid‐infrared graphene‐metal hybrid metasurfaces, it also provides guidelines for improving the efficiency of hybrid optical systems operating across a broad spectral range. Examples of potential applications encompass advanced optical modulators,^[^ ^66^, ^73^, ^74^ ^]^ SERS substrates,^[^ ^75^, ^76^ ^]^ and spatial light modulators.^[^ ^77^, ^78^, ^79^, ^80^, ^81^ ^]^

To mitigate ion‐induced damage in the Al_2_O_3_ etch stop layer, future work could focus on optimizing milling and ALD parameters.^[^ ^82^, ^83^ ^]^ This includes reducing the beam current and acceleration voltage, tuning the ALD temperature to enhance film uniformity and interface quality,^[^ ^84^ ^]^ applying H_2_ plasma cleaning prior to deposition, and performing post‐deposition annealing to reduce defects.^[^ ^85^, ^86^, ^87^, ^88^ ^]^ Additionally, research could aim to improve the quality of the metallic nanofilm by using ALD metal deposition instead of sputtering to minimize edge roughness. Gate tunability may also be further enhanced by implementing a top‐gated GFET with a low equivalent oxide thickness (EOT).

## 4 Experimental Section

### GFET Fabrication and Characterization

A 300 mm p‐doped silicon wafer was used to create a Fabry‐Perot cavity with a metal/dielectric/graphene/dielectric/metal stack consisting of Au (30 nm)/Pd (5 nm), Al_2_O_3_ (4 nm), graphene, Al_2_O_3_ (300 nm)/Si_3_N_4_ (15 nm), and Cu (250 nm). The 300 nm Al_2_O_3_ layer was selected based on FDTD simulations to maximize absorption efficiency ≈8 µm.^[^ ^89^ ^]^ To fabricate the cavity, an 800 nm Cu layer was first deposited and subsequently planarized to a final thickness of 250 nm using chemical–mechanical polishing (CMP), resulting in atomic‐scale surface roughness. A relatively thick Cu layer (250 nm) was chosen for several fabrication‐related reasons. First, CMP from an initial thickness of 800 nm down to 250 nm is a well‐established and controllable process in CMOS back‐end fabrication.^[^ ^90^, ^91^ ^]^ Second, thicker Cu films generally produce larger grain sizes, which can reduce optical losses. Third, the increased thickness enhances mechanical stability and ensures robust mid‐IR blocking. The Cu deposition and polishing strategy is consistent with established practices in high‐quality metal film preparation.^[^ ^90^ ^]^

A 15 nm Si_3__4_ layer was deposited by CVD on top of the Cu layer. The Si_3_N_4_ acts as a seeding layer, enhancing the nucleation and uniformity of the subsequent ALD‐grown Al_2_O_3_ layer (300 nm at 250 °C, Cambridge NanoTech Savannah). As demonstrated in previous work, the Si_3_N_4_ seeding layer significantly improves the surface quality of the dielectric stack, which facilitates graphene transfer and improves device performance.^[^ ^89^ ^]^

Commercial CVD‐grown SLG (Trivial Transfer Graphene, ACS Materials Co., MA) was employed. After dissolving the sacrificial layer by immersing the product in water, the PMMA‐supported SLG was transferred onto the substrate. The graphene channel was patterned using e‐beam lithography, and the excessive graphene was removed via oxygen‐based reactive ion etching, resulting in a rectangular graphene channel.

To promote precursor adsorption onto the non‐reactive graphene surface, a low‐temperature Trimethylaluminum Physiosorbed‐Precursor‐Assisted ALD process was employed in a 300 mm ALD system (Polygon8300, ASM) to grow, a 1–2 nm Al_2_O_3_ interfacial layer.^[^ ^92^ ^]^ To facilitate a high‐temperature (250°C) Al_2_O_3_ growth, a subsequent 2 nm Al_2_O_3_ layer was deposited using a cyclic TMA‐H_2_O ALD process in another 300 mm ALD system (XP4, ASM). To remove excess Al_2_O_3_ from the Pd/Au top layer, BCl_3_ and Cl_2_‐based inductively coupled plasma (ICP) etching was performed, followed by the deposition of a 200 nm Au layer with a 10 nm Pd adhesion layer for electrical contacts. Finally, electrical transport properties were measured using a Keithley 2450A nanovoltmeter under a DC current of 1 µA, with the back‐gate voltage applied from a Keithley 2400 voltage source.

### Fabrication of Sub‐10 nm Nanogaps

The LOM devices, consisting of a 30 nm thick Au layer with a 5 nm Pd adhesion layer, were fabricated using a similar lithography process but developed in a 0°C MIBK: IPA (1:3) solution to increase resolution and produce sharper antenna edges (see details in Section SII, Supporting Information). The IMM devices were obtained after an additional deposition and etching step. First, a 15 nm conformal Au layer was sputtered using a commercial sputtering system (Lab 18 Thin Film Deposition System, Kurt J. Lesker Co.), followed by Xe ion milling with 90 mA beam current and 24 V acceleration voltage.

### Optical Spectrum Measurement

Samples were characterized under ambient conditions using a Fourier‐transform infrared (FTIR) spectrometer (Bruker Vertex 80v + Hyperion 2000) with a spectral resolution of 1 cm^−1^. The infrared light first passes through a KBr polarizer and is then focused onto the sample using a 15x Schwarzschild (Cassegrain) objective with a numerical aperture of 0.4. A polarizer was used to assure excitation along the long axis of the nanoantennas.

The reflected signal is detected using a mercury cadmium telluride detector, cooled with liquid nitrogen to enhance sensitivity in the infrared range. The reflectance spectra were normalized to a 200 nm thick gold film deposited on the same Al_2_O_3_/Si_3_N_4_/Cu substrate for reference.

## Conflict of Interest

The authors declare no conflict of interest.

## Author Contributions

E.J. and N.V. initiated and supervised the research. F.H. performed the FDTD simulations, sample fabrication, graphene transfer, electrical and optical measurements. F.H., E.J., K.P., and J.V.d.V were involved in the preparation of the experimental set‐up to perform automatic GFET electrical transport measurements and related data analysis. F.H., Z.L., and H.T. performed the ALD deposition and substrate preparation. F.H., K.P., X.Z., E.J., and N.V. analyzed the results and prepared the manuscript. All authors reviewed the manuscript.

## Supporting information



Supporting Information

## Data Availability

The data that support the findings of this study are available in the supplementary material of this article.
