# Supplementary material for: Enhanced Spectral Tunability by Sub‐10 nm Nanogaps in Graphene‐Metal Hybrid Metasurfaces
Source: Adv Sci (Weinh). 2025 Jul 30;12(40):e06898. doi: 10.1002/advs.202506898 (PMC12561178; doi:10.1002/advs.202506898)
Supplement: Supplementary file 1 — Supporting Information [file ADVS-12-e06898-s001.docx]

**Supplementary material for**

**Enhanced Spectral Tunability by Sub-10 nm Nanogaps in Graphene-Metal Hybrid Metasurfaces**

*Fei Han*^§†^*, Zaoyang Lin*^†║^*, Kacper Pilarczyk*^§‡^*, Hongwei Tang*^†§^*, Guy A. E. Vandenbosch***, Joris Van de Vondel*^§^*,* *Xuezhi Zheng***^⁂^, Niels Verellen*^†§^*, Ewald Janssens*^§^

^§^Quantum Solid-State Physics, Department of Physics and Astronomy, KU Leuven, Celestijnenlaan 200D, 3001 Leuven, Belgium

‎^†^IMEC, Kapeldreef 75, 3001 Leuven, Belgium

^║^Department of Chemistry, KU Leuven, Celestijnenlaan 200F, 3001 Leuven, Belgium

^‡^Faculty of Physics and Applied Computer Science, AGH University of Science and Technology,
al. A. Mickiewicza 30, 30-059 Kraków, Poland

*WaveCoRE research group, ESAT, KU Leuven, Kasteelpark Arenberg 10, 3001 Leuven, Belgium

^⁂^Polariton-driven Light-Matter Interactions (POLIMA), University of Southern Denmark, Campusvej 55, 5230 Odense, Denmark

AUTHOR ADDRESS: [xuezhi.zheng@esat.kuleuven.be](mailto:xuezhi.zheng@esat.kuleuven.be); [ewald.janssens@kuleuven.be](mailto:ewald.janssens@kuleuven.be)

**Contents:**

I: Optical characterization of the metasurface with different gap sizes………………………………2

II: Fabrication details ………………………………………………………………………...………3

III: Characterization of pinholes caused by ion milling.…………………………………………...…4

IV: Simulation of the active reflectance tuning of the mid-infrared metasurface.…….…………...…6

References ……………………………………………………………………………........…………8

**I: Optical characterization of the metasurface with different shapes and gap sizes**

The optical properties of the fabricated metasurfaces were investigated by recording reflection spectra in the 4-10 µm wavelength range. Metasurfaces with antenna arrays of different geometries and gap sizes *g* were examined. **Figure S1a, S1b** and **S1c** show the measured reflectance of LOM devices with rectangular, ‘I’-shaped, and triangular antennas, respectively. The corresponding IMM devices with reduced gap sizes, obtained after adding a 15 nm PVD gold layer and subsequent ion-milling, are shown in **Figure S1d, S1e** and **S1f**. SEM images in the insets illustrate the smallest and largest measured gaps for the different shapes.


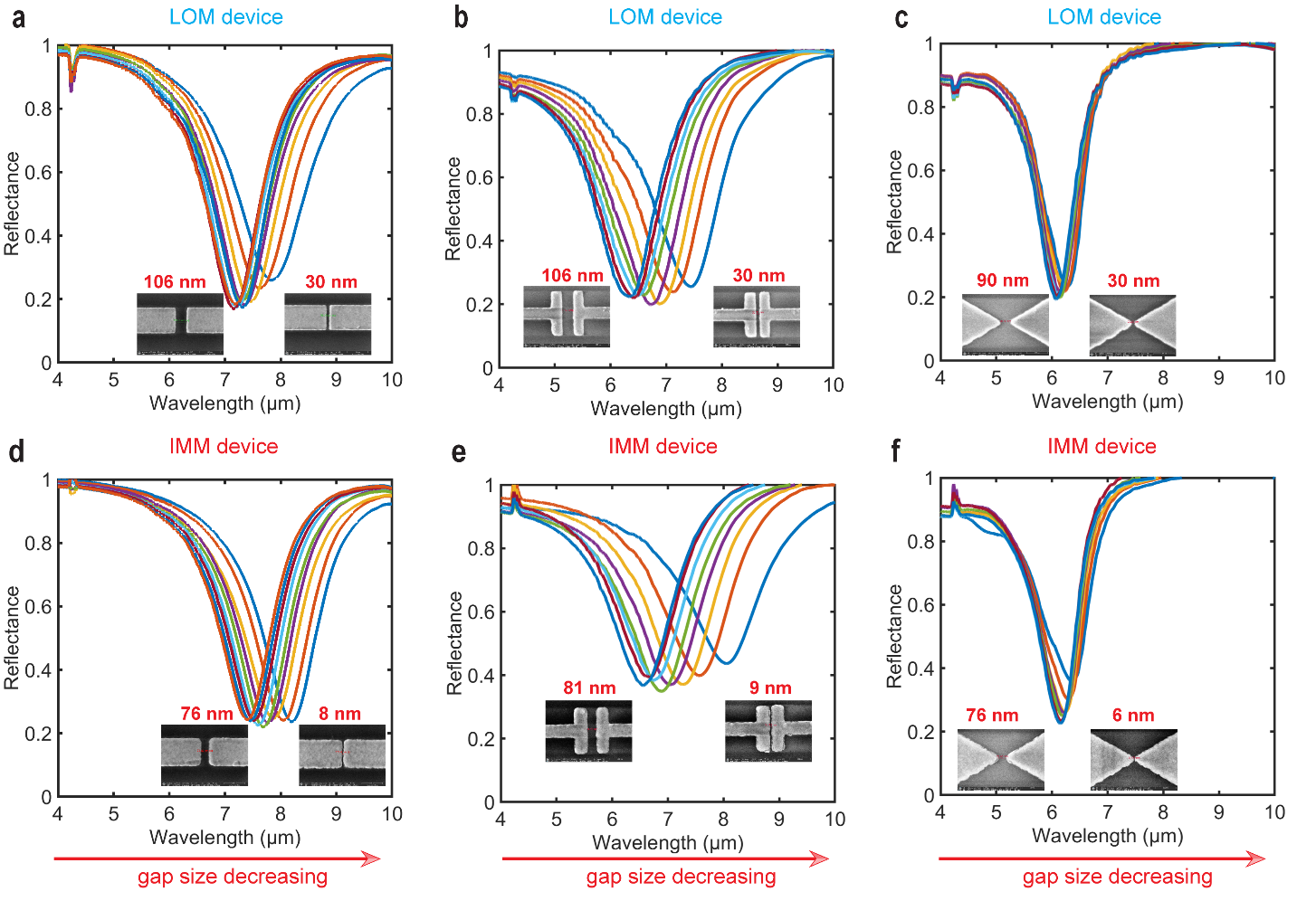


***Figure S1.*** *Experiments on fabricated metasurfaces with different shapes and gap sizes. Measured reflectance spectra of (a) (b) (c) LOM and (b) (e) (f) IMM devices. The thicknesses of Pd/Au are equal to 5 nm/30 nm and the antenna length was fixed to 2 µm. The spectra shift to the red with decreasing gap size. The measured gap sizes of the LOM (IMM) devices vary for rectangular shapes from 106 to 30 nm (76 to 8 nm), for ‘I’-shapes* *from 106 to 30 nm (81 to 9 nm),* *and for triangular shapes from 90 to 30 nm (76 to 7 nm). The nominal gaps decrease with a step size of 10 nm.*

**Figure S1** shows for all morphologies a red shift in the resonance wavelength with decreasing gap size. This trend is fully consistent with theoretical predictions,^1–3^ and can be attributed to the enhanced plasmonic coupling between the paired antennas as the gap becomes narrower.^1,4–6^ The measured spectral shift is therefore a reliable means to confirm the scaling of nanogaps.

**II: Fabrication details**

Low-temperature development was employed to enhance the quality of the patterned structure. Specifically, after patterning via electron beam lithography (EBL, Raith GmbH), the sample was developed at 0°C in a 3:1 isopropyl alcohol (IPA) to methyl isobutyl ketone (MIBK) solution for 30 seconds. The low temperature improves the resolution of the process compared to room-temperature development^7–9^, enabling the fabrication of rectangular antennas with sharp edges, which is crucial for achieving sub-10 nm gaps. Upon removal from the cold developer, the sample was continuously blown with N₂ for 60 seconds to stop the development and prevent frosting due to the low temperature.

As described in the main text, the fabrication of the metasurface with sub-10 nm gaps involves additional deposition and etching steps. A 15 nm conformal Au layer was deposited via sputtering using a commercial system (Lab 18 Thin Film Deposition System, Kurt J. Lesker Co.), followed by Xe ion milling. The selection of the ion milling etching time is important as the excessive film needs to be removed completely without damaging the graphene.


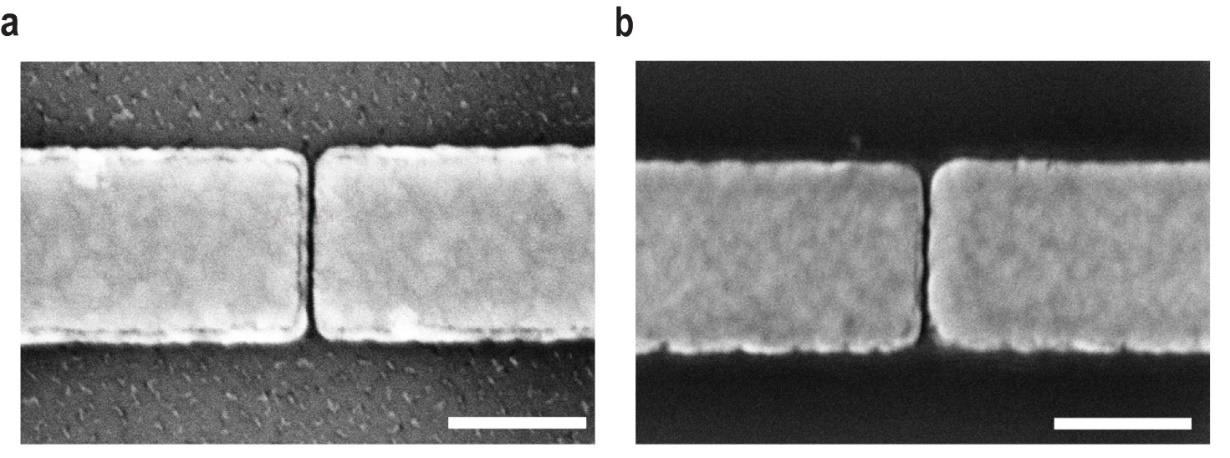


***Figure S2****. Zoomed-in SEM images at 250,000× magnification of fabricated sub-10 nm gap metasurfaces after ion milling for (a) 30 seconds and (b) 40 seconds. Scale bar: 200 nm.*

Two methods can be used to control the etching process. Either a calibration of the etch rate is made using a thicker film (e.g., a 100 nm gold film), whereafter an over-etching of the excessive film is done. Alternatively, SEM imaging is used to check if the etch process is complete. Incomplete etching goes together with the presence of residual gold particles. In this study, the second approach was chosen to preserve the Al₂O₃ layer and minimize unwanted ion-induced doping in graphene. As shown in **Figure S2a** and **S2b**, gold particles are present around the metal antennas after 30 seconds of ion milling, whereas those particles are completely removed after etching for 40 seconds. This approach provided the best estimation of the required etching time, while avoiding over-etching of the underlying Al₂O₃ layer.

**III: Characterization of defects caused by ion milling**

Standard top-view SEM imaging has limited capability in revealing subtle morphological changes induced by ion milling. To better visualize surface changes around antennas, 50° tilted SEM images were acquired for both LOM (**Figure S3a**) and IMM (**Figure S3b**) devices. In the LOM device, randomly distributed surface defects are visible as black dots, primarily located in the exposed regions between the antenna arrays. These may result from variations in surface morphology due to imperfect coverage of Al_2_O_3_ layer grown by ALD, contamination, or pre-existing imperfections in the graphene. Similar surface features earlier have been observed in ALD-grown films, particularly if the nucleation is non-uniform.^10,11^ In contrast, the IMM device shows a higher density of surface defects, which may result from damage or defect formation in the Al₂O₃ layer caused by the ion milling process. As seen in **Figure** **S2a** and **S3b**, Au residues appear as bright white dots sitting on black defects, producing a higher contrast than the surrounding holes.

The increase in the amount of surface defects is confirmed by Atomic Force Microscopy (AFM). **Figure S4** shows tapping-mode topographic images of the LOM (**Figure S4a**) and IMM (**Figure S4b**) devices. The LOM image displays a relatively smooth surface with sparse nanoscale features, while the IMM image reveals a higher density of pits distributed across the scanned area. These nanoscale irregularities are consistent with localized damage or void formation in the Al₂O₃ layer. Since the AFM detects topographical variations rather than material contrast, the increased roughness observed in the IMM device is more likely due to physical damage from ion milling and the adsorption of ambient particles, which are more clearly identified in SEM images. Together, the SEM and AFM results indicate that the surface irregularities in the IMM device primarily originate from ion milling–induced damage or defects in the Al₂O₃ layer. This highlights the importance of optimizing the fabrication process to minimize such damage.


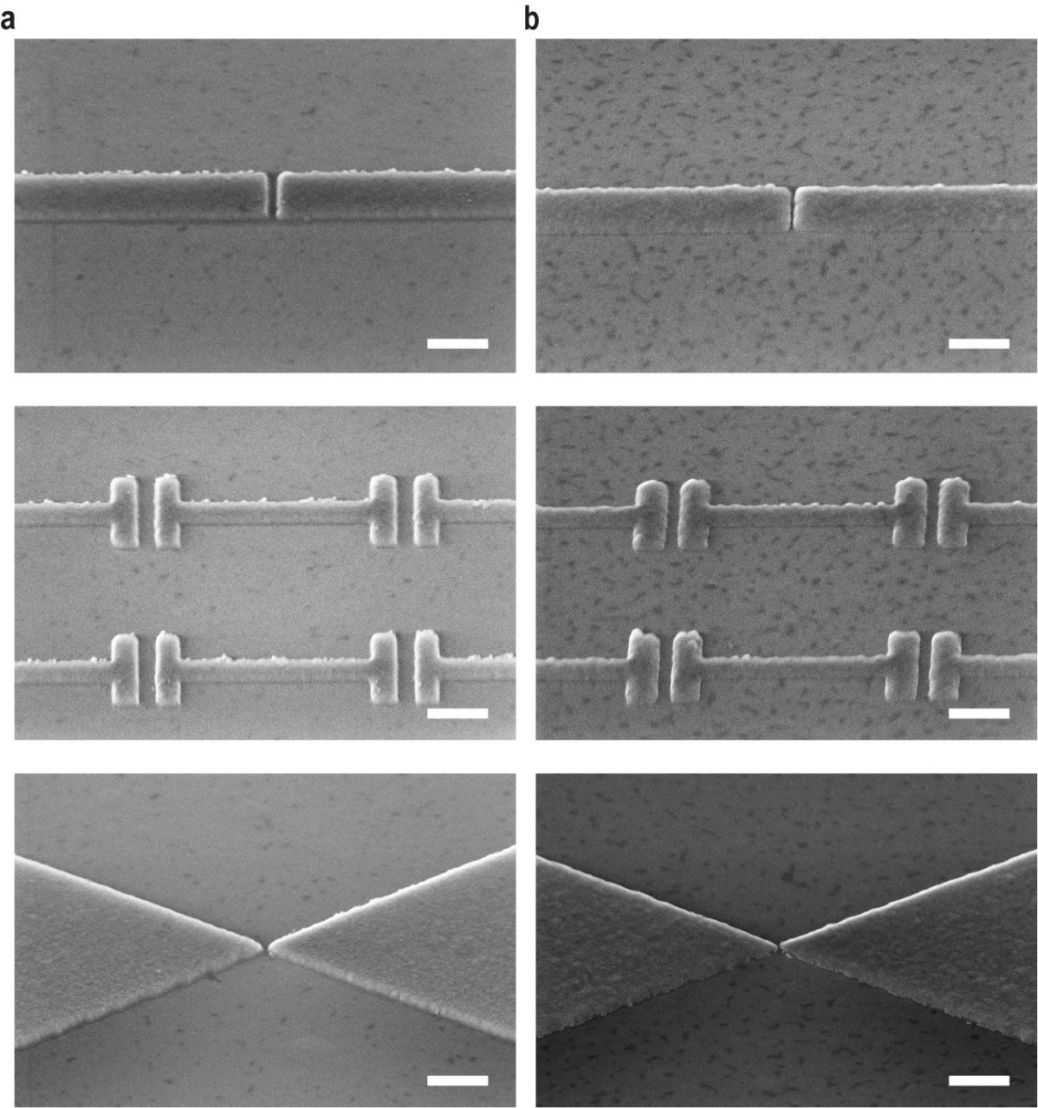


***Figure S3.*** *50° tilted SEM images at 100,000× magnification of (a) the LOM device and (b) the IMM device in different morphologies: rectangular-shaped (top), ‘I’-shaped (middle), and triangular-shaped (bottom) gold antenna arrays. Scale bar: 250 nm.*


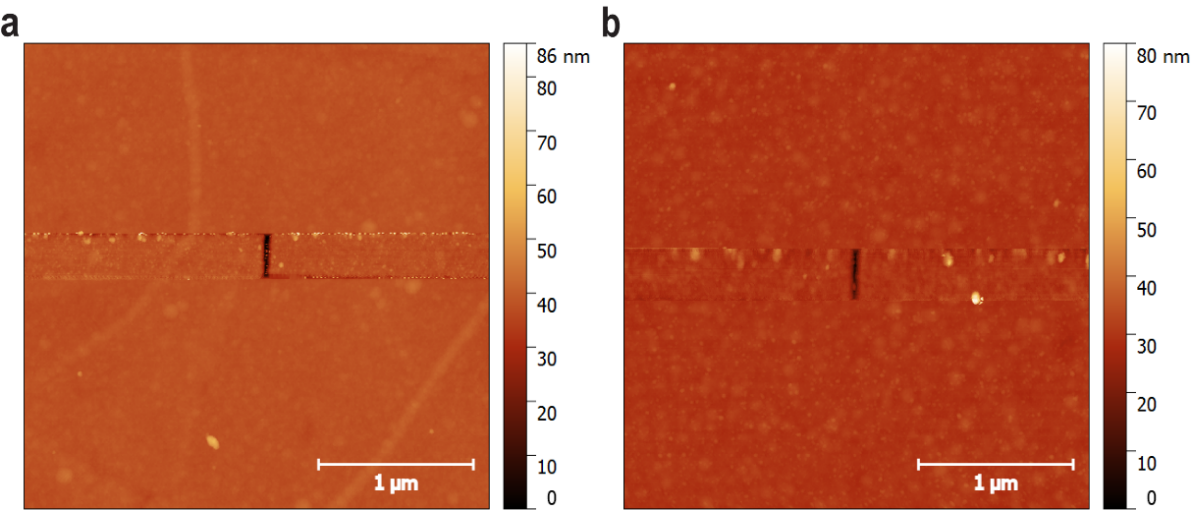


***Figure S4.*** *Tapping-mode AFM images of (a) the LOM device and (b) the IMM device. Both devices exhibit nanoscale surface defects, but the IMM device shows a higher roughness and more defects.*

**IV: Simulation of the active reflectance tuning of the mid-infrared metasurface**

To investigate enhanced tunability for mid-infrared modulators application, we performed FDTD simulations of the reflectance spectra for the metasurfaces with gap sizes of 100 nm, 30 nm, 80 nm and 10 nm. **Figure S5** presents the simulated spectra corresponding to Fermi energies from 0 to 0.25 eV. The relation between the Fermi energy in the simulations and the applied gate voltage in the experiment is estimated based on charge carrier concentrations, as explained in our previous work.^12^ The selected E_F_ range of 0 to 0.25 eV is expected to correspond to a gate voltage range of ±80 V. The Fermi energies’ value is estimated from the gate voltage using the equation:^12^

$E_{F}=\hbar\nu_{F}\sqrt{\frac{\pi C_{g}\left| V_{g}-V_{CNP} \right|}{e}}$, (1)

where *C_g_* is the areal gate capacitance and the Fermi velocity *v_F_* is assumed to be 10^6^ m/s.^13^

The FDTD simulations show that the resonant wavelength of all four devices exhibits a clear blue shift with increasing Fermi energy, with the shift being more pronounced for smaller gap sizes. For example, for the 30 nm gap device, the simulation predicts a resonance shift from 8.5 µm to 7.8 µm as E_F_ increases from 0 to 0.25 eV, in good agreement with the experimental shift from 7.9 µm to 7.4 µm.


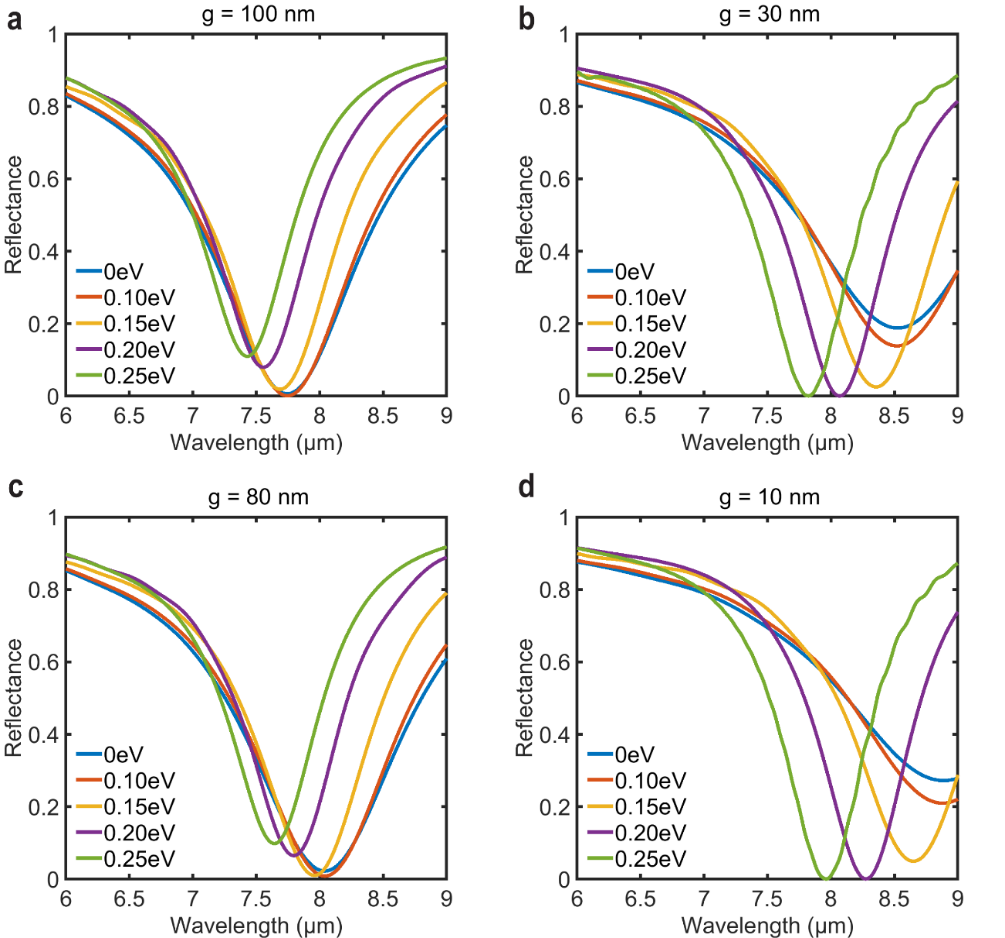


***Figure S5.*** *Simulated reflectance spectra of metasurfaces with gap size of (a) g = 100 nm, (b) g = 30 nm, (c) g = 80 nm, and (d) g = 10 nm for different values of the Fermi energy of graphene in the 0 to 0.25 eV range.*

The change in reflectance and the simulated MD as a function of wavelength are extracted from the FDTD simulations, which is presented in **Figure S6** for the metasurfaces with 30 nm and 10 nm gaps. When the incident wavelength lies on the blue side of the original resonance, increasing the graphene Fermi energy enhances (reduces) the optical absorption (reflectance). In contrast, on the red side of the resonance, the reflectance increases with the Fermi energy. The FDTD simulated MD in the blue side reaches a maximum of 100%. This is because the cavity in the FDTD simulation is designed for near-perfect absorption, whereas in practice the absorption is lower due to material and fabrication limitations.


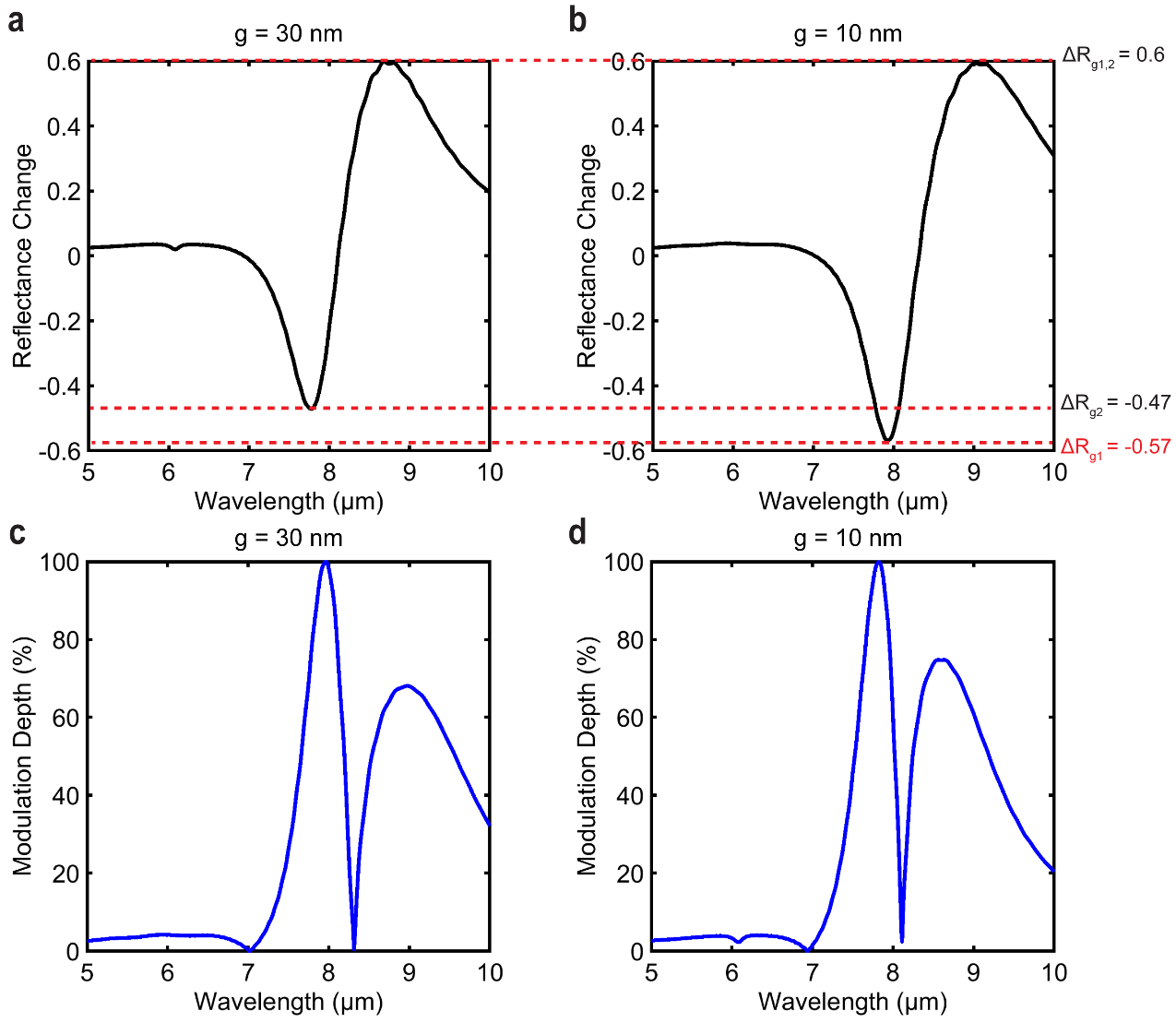


***Figure S6.*** *Extracted reflectance change for the OFF-ON transition, where the ON (OFF) state corresponds to FDTD simulation at E_F_ = 0 V (E_F_ = 0.25 eV) of metasurface with gap size of (a) g = 30 nm and (b) g = 10 nm as a function of the wavelength.* *The maximal reflectance decrease changes from 0.47 to 0.57 after the gap size is reduced from 30 nm to 10 nm. Extracted reflectance modulation depth for the OFF-ON transition for the metasurfaces with gap size of (c) g = 30 nm and (d) g = 10 nm as a function of the wavelength.*

REFERENCES

1. Prodan, E., Radloff, C., Halas, N. J. & Nordlander, P. A hybridization model for the plasmon response of complex nanostructures. *Science* **302**, 419–422 (2003).

2. Duan, H., Fernández-Domínguez, A. I., Bosman, M., Maier, S. A. & Yang, J. K. W. Nanoplasmonics: Classical down to the Nanometer Scale. *Nano Lett.* **12**, 1683–1689 (2012).

3. Choi, S. *et al.* Near- and far-field study of polarization-dependent surface plasmon resonance in bowtie nano-aperture arrays. *Opt. Express* **31**, 31760–31767 (2023).

4. Cubukcu, E. *et al.* Plasmonic laser antennas and related devices. *IEEE J. Sel. Top. Quantum Electron.* **14**, 1448–1461 (2008).

5. M. O. El-Shenawee. Polarization Dependence of Plasmonic Nanotoroid Dimer Antenna. *IEEE Antennas Wireless Propag. Lett.* **9**, 463–466 (2010).

6. Lin, L. & Zheng, Y. Optimizing plasmonic nanoantennas via coordinated multiple coupling. *Sci. Rep.* **5**, 14788 (2015).

7. Yasin, S., Hasko, D. G. & Ahmed, H. Comparison of MIBK/IPA and water/IPA as PMMA developers for electron beam nanolithography. *Microelectron. Eng.* **61–62**, 745–753 (2002).

8. Hu, W. (Walter), Sarveswaran, K., Lieberman, M. & Bernstein, G. H. Sub-10 nm electron beam lithography using cold development of poly(methylmethacrylate). *J. Vac. Sci. Technol. B* **22**, 1711–1716 (2004).

9. Koh, A. L., Fernández-Domínguez, A. I., McComb, D. W., Maier, S. A. & Yang, J. K. W. High-Resolution Mapping of Electron-Beam-Excited Plasmon Modes in Lithographically Defined Gold Nanostructures. *Nano Lett.* **11**, 1323–1330 (2011).

10. Prabhu, B., Suryanarayana, C., An, L. & Vaidyanathan, R. Synthesis and characterization of high volume fraction Al–Al2O3 nanocomposite powders by high-energy milling. *Mater. Sci. Eng. A.* **425**, 192–200 (2006).

11. Yang, Q., Kalathiparambil, K., Elg, D. T., Ruzic, D. & Kriven, W. M. Microstructural damage of α-Al2O3 by high energy density plasma. *Acta Mater.* **132**, 479–490 (2017).

12. Han, F. *et al.* Tunable Mid-Infrared Multi-Resonant Graphene-Metal Hybrid Metasurfaces. *Adv. Opt. Mater.* **12**, 2303085 (2024).

13. Novoselov, K. S. *et al.* Electric Field Effect in Atomically Thin Carbon Films. *Science* **306**, 666–669 (2004).
